# 3-*tert*-Butyl-1-(3-nitro­phen­yl)-1*H*-pyrazol-5-amine

**DOI:** 10.1107/S1600536812042791

**Published:** 2012-10-20

**Authors:** Simón Hernández-Ortega, Fernando Cuenú-Cabezas, Rodrigo Abonia-González, Armando Cabrera-Ortiz

**Affiliations:** a Instituto de Química, Universidad Nacional Autónoma de México, circuito exterior, ciudad universitaria, México 04510, México; b Laboratorio de Química Inorgánica y Catálisis, Programa de Química, Universidad del Quindio, Avenida Bolivar Calle 12 Norte, Armenia, Colombia; c Departamento de Química, Universidad del Valle, A. A. 25360, Cali, Colombia

## Abstract

In the title compound, C_13_H_16_N_4_O_2_, the pyrazole ring forms a dihedral angle of 50.61 (6)° with the 3-nitro-phenyl ring. The plane of the nitro group is twisted by 6.8 (7)° out of the plane of the phenyl ring. In the crystal, the mol­ecules are linked by N—H⋯N and N—H⋯O hydrogen bonds, forming sheets in the *bc* plane. In addition, a weak C—H⋯N inter­action is observed.

## Related literature

For background to pyrazole-based ligands, see; Ahmed *et al.* (2005 ▶); Abonia *et al.* (2002 ▶, 2004 ▶, 2010 ▶); Guerrero *et al.* (2009 ▶); Quiroga *et al.* (2008 ▶); Schutznerová, *et al.* (2012 ▶). For structure of an isomer of the title compound, see: Low *et al.* (2004 ▶).
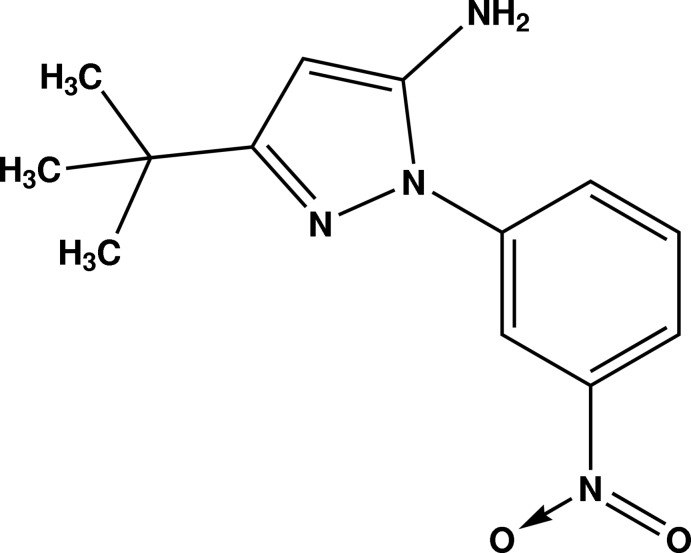


## Experimental

### 

#### Crystal data

C_13_H_16_N_4_O_2_
*M* *_r_* = 260.30
Monoclinic, 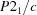

*a* = 11.9421 (14) Å
*b* = 9.6419 (11) Å
*c* = 11.7694 (13) Å
β = 93.504 (2)°
*V* = 1352.6 (3) Å^3^
*Z* = 4
Mo *K*α radiation
μ = 0.09 mm^−1^
*T* = 298 K
0.46 × 0.36 × 0.32 mm

#### Data collection

Bruker SMART APEX CCD area-detector diffractometer
14529 measured reflections
2486 independent reflections
2036 reflections with *I* > 2σ(*I*)
*R*_int_ = 0.044

#### Refinement

*R*[*F* ^2^ > 2σ(*F* ^2^)] = 0.040
*wR*(*F* ^2^) = 0.115
*S* = 1.05
2486 reflections
181 parameters
2 restraints
H atoms treated by a mixture of independent and constrained refinement
Δρ_max_ = 0.17 e Å^−3^
Δρ_min_ = −0.17 e Å^−3^

### 

Data collection: *SMART* (Bruker, 1999 ▶); cell refinement: *SAINT* (Bruker, 1999 ▶); data reduction: *SAINT*; program(s) used to solve structure: *SHELXTL* (Sheldrick, 2008 ▶); program(s) used to refine structure: *SHELXTL*; molecular graphics: *SHELXTL*; software used to prepare material for publication: *SHELXTL*.

## Supplementary Material

Crystal structure: contains datablock(s) I, global. DOI: 10.1107/S1600536812042791/bt6847sup1.cif

Structure factors: contains datablock(s) I. DOI: 10.1107/S1600536812042791/bt6847Isup2.hkl

Additional supplementary materials:  crystallographic information; 3D view; checkCIF report

## Figures and Tables

**Table 1:** Hydrogen-bond geometry (Å, °)

*D*—H⋯*A*	*D*—H	H⋯*A*	*D*⋯*A*	*D*—H⋯*A*
N4—H4*A*⋯N2^i^	0.90 (1)	2.23 (1)	3.1195 (17)	172 (2)
N4—H4*B*⋯O1^ii^	0.90 (1)	2.39 (1)	3.241 (2)	160 (2)
C14—H14⋯N4^iii^	0.93	2.54	3.403 (2)	155

Symmetry codes: (i) 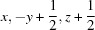; (ii) 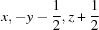; (iii) 
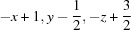
.

**Fig. 1.**
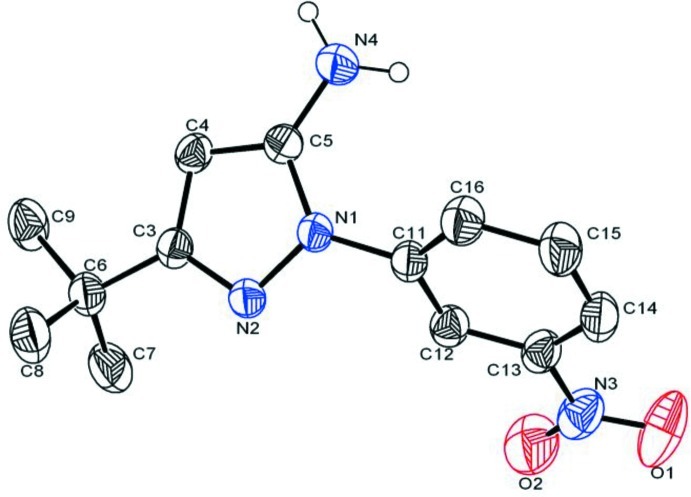
Structure of (I), with the numbering scheme. The displacement ellipsoids are drawn to 40% of probability.

**Fig. 2.**
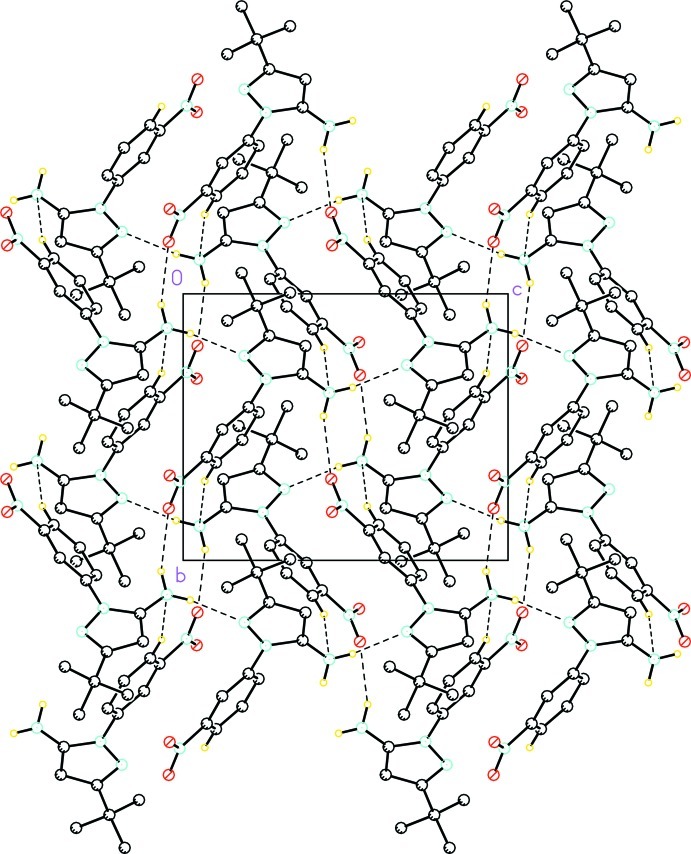
The crystal packing of (I), only the H atoms involved in intermolecular interaction were drawn.

## supplementary crystallographic information

### Comment

The recent past has evidenced an ever-increasing interest in pyrazole based ligands. The interest in such compounds is due, first of all, to their variety of coordination complexes with a great number of metal ions and, second, to their ability to provide an extensive variety of coordination geometries and significant structural nuclearity when introducing different kinds of heteroatoms (Ahmed *et al.* 2005; Schutznerová *et al.* 2012). The past few years have seen considerable rise in interest in the design of various pyrazole-based ligands for particular metal binding site (Guerrero *et al.* 2009).

As a part of our current research work focused on the development of new bioactive heterocyclic compounds and continuing with the use of pyrazolic Schiff bases (Quiroga *et al.*, 2008; Abonia *et al.* 2002, 2004, 2010), in the synthesis of pyrazolopyrimidines, we want to describe the compound 5-amino-3-*tert*-butyl-1-(3-nitro-phenyl)-1*H*-pyrazole (I), which is a structural isomer of a related compound previously reported by Low (Low *et al.*, 2004).

The structure of the title compound is shown in Figure 1. The compound consists of a ring pyrazole substituted by 3-nitro-phenyl ring bonded to N1, amino group in C5 and *tert*butyl group in C3. The pyrazole and phenyl rings are not coplanar, they are forming a dihedral angle of 50.61 (6)°. The nitro group is rotated around C14—N3 bond by 6.8 (3)°. These angle values are larger than those described for the isomeric compound 5-amino-3-*tert*butyl-1-(4-nitro-phenyl)-1*H*-pyrazole (Low *et al.*, 2004). In the crystal, the molecules are linked by N—H–N and N—H–O intermolecular hydrogen bonds forming sheets in the *bc* plane. In additon, a weak intermolecular C-H···N interaction is observed (Figure 2, Table 1).

### Experimental

To a solution of conc hydrochloric acid (3.8 ml) in water (33 ml), 3-nitrophenylhydrazine (1.5001 g, 9.87 mmol) and 4,4-dimethyl-3-oxopentanenitrile (1.8502 g, 14.80 mmol) were added. The mixture was heated at 70 °C for 1 h. Then, conc hydrochloric acid (3.8 ml) was added and the mixture was heated for 1 h more. After cooling, crushed ice was added and neutralized with conc ammonium hydroxide. The resulting solid was filtered under reduced pressure, washed with cold water (3 *X* 5 ml) and dried at ambient temperature affording the title compound (I) as a yellow solid [yield 1.744 g, 68%, m.p. 375 K]. MS (70 eV) m/*z* (%): 260 (55), 245 (100), 218 (88), 190 (73). Anal. Calc. for C_13_H_16_N_4_O_2_; C 59.99; H 6.20; N 21.52%, found C 60.36; H 6.42; N 21.88%. Crystals of the title compound suitable for single-crystal X-ray diffraction were grown by slow diffusion of pentane into a CH_2_Cl_2_ solution of the title compound.

### Refinement

The positional parameters of the amino H atom were refined with a distance restraint of 0.90 (1)Å while those of the other H atoms were calculated geometrically (C—H = 0.93–0.98 Å). All H atoms were refined with *U*_iso_(H) = 1.2U_eq_ of the parent atom.

### Crystal data

**Table:**

C_13_H_16_N_4_O_2_	*F*(000) = 552
*M**_r_* = 260.30	*D*_x_ = 1.278 Mg m^−^^3^
Monoclinic, *P*2_1_/*c*	Mo *K*α radiation, λ = 0.71073 Å
Hall symbol: -P 2ybc	Cell parameters from 7587 reflections
*a* = 11.9421 (14) Å	θ = 2.7–25.3°
*b* = 9.6419 (11) Å	µ = 0.09 mm^−^^1^
*c* = 11.7694 (13) Å	*T* = 298 K
β = 93.504 (2)°	Prism, orange
*V* = 1352.6 (3) Å^3^	0.46 × 0.36 × 0.32 mm
*Z* = 4	

### Data collection

**Table:**

Bruker SMART APEX CCD area-detector diffractometer	2036 reflections with *I* > 2σ(*I*)
Radiation source: fine-focus sealed tube	*R*_int_ = 0.044
Graphite monochromator	θ_max_ = 25.4°, θ_min_ = 2.7°
Detector resolution: 0.83 pixels mm^-1^	*h* = −14→14
ω scans	*k* = −11→11
14529 measured reflections	*l* = −14→14
2486 independent reflections	

### Refinement

**Table:**

Refinement on *F*^2^	Primary atom site location: structure-invariant direct methods
Least-squares matrix: full	Secondary atom site location: difference Fourier map
*R*[*F*^2^ > 2σ(*F*^2^)] = 0.040	Hydrogen site location: inferred from neighbouring sites
*wR*(*F*^2^) = 0.115	H atoms treated by a mixture of independent and constrained refinement
*S* = 1.05	*w* = 1/[σ^2^(*F*_o_^2^) + (0.0644*P*)^2^ + 0.1233*P*] where *P* = (*F*_o_^2^ + 2*F*_c_^2^)/3
2486 reflections	(Δ/σ)_max_ < 0.001
181 parameters	Δρ_max_ = 0.17 e Å^−^^3^
2 restraints	Δρ_min_ = −0.17 e Å^−^^3^

### Special details

**Table:**

Geometry. All e.s.d.'s (except the those in the dihedral angle between two l.s. planes) are estimated using the full covariance matrix. The cell e.s.d.'s are taken into account individually in the estimation of e.s.d.'s in distances, angles and torsion angles; correlations between e.s.d.'s in cell parameters are only used when they are defined by crystal symmetry. An approximate (isotropic) treatment of cell e.s.d.'s is used for estimating e.s.d.'s involving l.s. planes.
Refinement. Refinement of *F*^2^ against ALL reflections. The weighted *R*-factor *wR* and goodness of fit *S* are based on *F*^2^, conventional *R*-factors *R* are based on *F*, with *F* set to zero for negative *F*^2^. The threshold expression of *F*^2^ > σ(*F*^2^) is used only for calculating *R*-factors(gt) *etc*. and is not relevant to the choice of reflections for refinement. *R*-factors based on *F*^2^ are statistically about twice as large as those based on *F*, and *R*- factors based on ALL data will be even larger.

### Fractional atomic coordinates and isotropic or equivalent isotropic displacement parameters (Å^2^)

**Table:**

	*x*	*y*	*z*	*U*_iso_*/*U*_eq_	
O1	0.61664 (14)	−0.30475 (15)	0.45577 (16)	0.1090 (6)	
O2	0.76468 (15)	−0.18160 (16)	0.45837 (14)	0.1006 (5)	
N1	0.71780 (9)	0.17992 (11)	0.75434 (9)	0.0403 (3)	
N2	0.78018 (9)	0.26705 (11)	0.68907 (9)	0.0426 (3)	
N3	0.67228 (15)	−0.20551 (15)	0.48900 (13)	0.0686 (4)	
N4	0.68987 (11)	0.12825 (14)	0.94996 (10)	0.0516 (3)	
H4A	0.7163 (13)	0.1491 (17)	1.0213 (9)	0.062*	
H4B	0.6699 (13)	0.0411 (11)	0.9321 (14)	0.062*	
C3	0.84041 (11)	0.34268 (13)	0.76494 (11)	0.0401 (3)	
C4	0.81984 (11)	0.30418 (14)	0.87650 (11)	0.0433 (3)	
H4	0.8523	0.3421	0.9433	0.052*	
C5	0.74255 (11)	0.19985 (13)	0.86760 (11)	0.0400 (3)	
C6	0.91855 (12)	0.45492 (15)	0.72699 (13)	0.0488 (4)	
C7	0.99208 (17)	0.3989 (2)	0.63582 (17)	0.0768 (6)	
H7A	1.0374	0.3241	0.6669	0.115*	
H7B	1.0398	0.4716	0.6110	0.115*	
H7C	0.9453	0.3657	0.5723	0.115*	
C8	0.85048 (16)	0.57837 (16)	0.67990 (16)	0.0698 (5)	
H8A	0.8051	0.5500	0.6138	0.105*	
H8B	0.9005	0.6509	0.6594	0.105*	
H8C	0.8030	0.6119	0.7368	0.105*	
C9	0.99367 (15)	0.50386 (18)	0.82913 (16)	0.0657 (5)	
H9A	0.9483	0.5454	0.8845	0.098*	
H9B	1.0464	0.5709	0.8044	0.098*	
H9C	1.0334	0.4260	0.8626	0.098*	
C11	0.64947 (11)	0.07495 (13)	0.70143 (11)	0.0413 (3)	
C12	0.69297 (12)	−0.00946 (14)	0.62034 (12)	0.0458 (4)	
H12	0.7658	0.0027	0.5985	0.055*	
C13	0.62517 (13)	−0.11245 (14)	0.57271 (12)	0.0490 (4)	
C14	0.51684 (14)	−0.13283 (15)	0.60126 (14)	0.0555 (4)	
H14	0.4727	−0.2023	0.5668	0.067*	
C15	0.47534 (14)	−0.04795 (17)	0.68205 (15)	0.0598 (4)	
H15	0.4022	−0.0603	0.7032	0.072*	
C16	0.54076 (12)	0.05548 (16)	0.73219 (13)	0.0535 (4)	
H16	0.5117	0.1124	0.7869	0.064*	

### Atomic displacement parameters (Å^2^)

**Table:**

	*U*^11^	*U*^22^	*U*^33^	*U*^12^	*U*^13^	*U*^23^
O1	0.1062 (12)	0.0705 (9)	0.1473 (15)	−0.0008 (8)	−0.0181 (10)	−0.0588 (10)
O2	0.1094 (13)	0.0912 (11)	0.1050 (12)	−0.0042 (9)	0.0366 (10)	−0.0347 (9)
N1	0.0438 (6)	0.0374 (6)	0.0390 (6)	−0.0066 (5)	−0.0035 (5)	0.0012 (5)
N2	0.0464 (7)	0.0400 (6)	0.0408 (6)	−0.0063 (5)	−0.0029 (5)	0.0030 (5)
N3	0.0837 (11)	0.0507 (8)	0.0697 (9)	0.0057 (8)	−0.0079 (8)	−0.0137 (7)
N4	0.0619 (8)	0.0505 (7)	0.0418 (7)	−0.0070 (6)	−0.0016 (6)	0.0068 (6)
C3	0.0411 (7)	0.0346 (7)	0.0435 (7)	0.0009 (6)	−0.0056 (6)	0.0008 (6)
C4	0.0487 (8)	0.0402 (7)	0.0398 (7)	−0.0025 (6)	−0.0089 (6)	−0.0019 (6)
C5	0.0429 (7)	0.0370 (7)	0.0393 (7)	0.0041 (6)	−0.0037 (6)	0.0025 (5)
C6	0.0518 (8)	0.0404 (7)	0.0530 (8)	−0.0076 (6)	−0.0062 (7)	0.0043 (6)
C7	0.0860 (13)	0.0648 (11)	0.0827 (13)	−0.0206 (10)	0.0292 (11)	0.0050 (10)
C8	0.0834 (12)	0.0428 (9)	0.0797 (12)	−0.0123 (8)	−0.0248 (10)	0.0123 (8)
C9	0.0610 (10)	0.0575 (10)	0.0756 (11)	−0.0208 (8)	−0.0191 (9)	0.0098 (8)
C11	0.0442 (8)	0.0361 (7)	0.0426 (7)	−0.0044 (6)	−0.0060 (6)	0.0030 (6)
C12	0.0460 (8)	0.0427 (8)	0.0478 (8)	−0.0014 (6)	−0.0044 (6)	0.0011 (6)
C13	0.0606 (9)	0.0365 (7)	0.0484 (8)	0.0008 (7)	−0.0084 (7)	−0.0012 (6)
C14	0.0612 (10)	0.0420 (8)	0.0611 (9)	−0.0129 (7)	−0.0132 (8)	0.0031 (7)
C15	0.0495 (9)	0.0606 (10)	0.0690 (10)	−0.0154 (8)	−0.0001 (8)	−0.0018 (8)
C16	0.0494 (9)	0.0525 (9)	0.0585 (9)	−0.0054 (7)	0.0020 (7)	−0.0050 (7)

### Geometric parameters (Å, º)

**Table:**

O1—N3	1.2159 (19)	C7—H7B	0.9600
O2—N3	1.204 (2)	C7—H7C	0.9600
N1—C5	1.3613 (17)	C8—H8A	0.9600
N1—N2	1.3858 (15)	C8—H8B	0.9600
N1—C11	1.4198 (16)	C8—H8C	0.9600
N2—C3	1.3297 (17)	C9—H9A	0.9600
N3—C13	1.470 (2)	C9—H9B	0.9600
N4—C5	1.3735 (18)	C9—H9C	0.9600
N4—H4A	0.901 (9)	C11—C12	1.380 (2)
N4—H4B	0.895 (9)	C11—C16	1.382 (2)
C3—C4	1.4005 (19)	C12—C13	1.378 (2)
C3—C6	1.5140 (19)	C12—H12	0.9300
C4—C5	1.3650 (19)	C13—C14	1.370 (2)
C4—H4	0.9300	C14—C15	1.370 (2)
C6—C8	1.526 (2)	C14—H14	0.9300
C6—C7	1.526 (2)	C15—C16	1.377 (2)
C6—C9	1.530 (2)	C15—H15	0.9300
C7—H7A	0.9600	C16—H16	0.9300
			
C5—N1—N2	111.43 (10)	C6—C8—H8A	109.5
C5—N1—C11	127.92 (11)	C6—C8—H8B	109.5
N2—N1—C11	120.20 (10)	H8A—C8—H8B	109.5
C3—N2—N1	104.32 (10)	C6—C8—H8C	109.5
O2—N3—O1	123.17 (17)	H8A—C8—H8C	109.5
O2—N3—C13	118.64 (15)	H8B—C8—H8C	109.5
O1—N3—C13	118.17 (17)	C6—C9—H9A	109.5
C5—N4—H4A	113.3 (11)	C6—C9—H9B	109.5
C5—N4—H4B	115.7 (11)	H9A—C9—H9B	109.5
H4A—N4—H4B	120.1 (16)	C6—C9—H9C	109.5
N2—C3—C4	111.44 (12)	H9A—C9—H9C	109.5
N2—C3—C6	120.78 (12)	H9B—C9—H9C	109.5
C4—C3—C6	127.78 (12)	C12—C11—C16	120.04 (13)
C5—C4—C3	106.26 (12)	C12—C11—N1	119.55 (12)
C5—C4—H4	126.9	C16—C11—N1	120.40 (13)
C3—C4—H4	126.9	C13—C12—C11	118.00 (13)
N1—C5—C4	106.53 (11)	C13—C12—H12	121.0
N1—C5—N4	122.60 (12)	C11—C12—H12	121.0
C4—C5—N4	130.77 (13)	C14—C13—C12	122.92 (14)
C3—C6—C8	109.90 (12)	C14—C13—N3	118.85 (14)
C3—C6—C7	110.23 (12)	C12—C13—N3	118.23 (14)
C8—C6—C7	109.71 (14)	C15—C14—C13	118.12 (14)
C3—C6—C9	109.33 (12)	C15—C14—H14	120.9
C8—C6—C9	108.57 (13)	C13—C14—H14	120.9
C7—C6—C9	109.07 (14)	C14—C15—C16	120.67 (15)
C6—C7—H7A	109.5	C14—C15—H15	119.7
C6—C7—H7B	109.5	C16—C15—H15	119.7
H7A—C7—H7B	109.5	C15—C16—C11	120.24 (14)
C6—C7—H7C	109.5	C15—C16—H16	119.9
H7A—C7—H7C	109.5	C11—C16—H16	119.9
H7B—C7—H7C	109.5		

### Hydrogen-bond geometry (Å, º)

**Table:**

*D*—H···*A*	*D*—H	H···*A*	*D*···*A*	*D*—H···*A*
N4—H4*A*···N2^i^	0.90 (1)	2.23 (1)	3.1195 (17)	172 (2)
N4—H4*B*···O1^ii^	0.90 (1)	2.39 (1)	3.241 (2)	160 (2)
C14—H14···N4^iii^	0.93	2.54	3.403 (2)	155

Symmetry codes: (i) *x*, −*y*+1/2, *z*+1/2; (ii) *x*, −*y*−1/2, *z*+1/2; (iii) −*x*+1, *y*−1/2, −*z*+3/2.
